# Dysphonia coping strategies, vocal handicap and anxiety/depression symptoms in pre and post-thyroidectomy patients

**DOI:** 10.1590/2317-1782/e20240302en

**Published:** 2025-11-17

**Authors:** Marcus Vinicius Conceição Gama, Gabriel Trevizani, Michelle Guimarães, Elma Heitmann

**Affiliations:** 1 Departamento de Fonoaudiologia, Universidade Federal do Espírito Santo – UFES - Vitória (ES), Brasil.; 2 Programa de Pós-graduação em Ciências da Saúde e Comunicação Humana, Universidade Estadual Paulista “Júlio de Mesquita Filho”- Unesp -Marília (SP), Brasil.

**Keywords:** Anxiety, Depression, Dysphonia, Thyroidectomy, Voice

## Abstract

**Purpose:**

To analyze coping strategies for dysphonia, vocal handicap, and anxiety/depression symptoms in patients undergoing thyroidectomy.

**Methods:**

A retrospective study with patients evaluated preoperatively, recently postoperatively, and three months post-thyroidectomy. Data from the Brazilian version of the Voice Disability Coping Questionnaire (B-VDCQ), Vocal Handicap Index (VHI), and Hospital Anxiety and Depression Scale (HADS) were analyzed. Patients with language, neurological, laryngeal, thyroid hormonal, or reflux disorders were excluded.

**Results:**

A total of 20 patients participated (mean age 54 years, SD±16.9), predominantly female (n=17; 85%), with partial thyroidectomy (n=14; 70%). In B-VDCQ, scores decreased between preoperative, recent postoperative, and three-month postoperative assessments, especially in the emotional focus domain, with no significant differences over time. In VHI, the organic domain had the highest scores, indicating mild vocal handicap without differences across assessments. In HADS, mild anxiety traits were observed preoperatively, reduced shortly after surgery, but increased after three months, while depression remained within normal limits. Preoperatively, a moderate positive correlation was found between depression and the organic and total VHI domains. In the recent postoperative period, anxiety showed a weak/moderate positive correlation with B-VDCQ and VHI. After three months, B-VDCQ had a moderate positive correlation with anxiety and total HADS, and a weak correlation between the "functional" and "organic" VHI domains and depression.

**Conclusion:**

Patients primarily use emotion-focused coping strategies, self-report mild vocal handicap, and exhibit mild anxiety traits preoperatively, which decrease postoperatively but increase after three months. The strongest correlations occurred in the immediate postoperative period.

## INTRODUCTION

The thyroid gland is one of the largest endocrine organs and has anatomical proximity to the recurrent laryngeal nerve, a branch of the vagus nerve, increasing the risk of injury during surgical access. It is responsible for the production of two hormones, thyroxine (T4) and triiodothyronine (T3), which act on practically all cells and are responsible for controlling various parts of the metabolism of the organs of the human body^(^1^)^ such as the control of heartbeat, intestinal peristalsis, regulation of the menstrual cycle, fertility, body temperature, weight control, mood, memory, cognitive function, and emotional control.

In the presence of benign/malignant nodules in the thyroid gland, there are different treatments such as monitoring, surgery, radioactive iodine therapy, hormone replacement, radiotherapy, and chemotherapy. In addition to the presence of nodules, structural aspects, size, location, and other factors are taken into consideration when surgery is indicated. Patients may develop changes in voice/swallowing and experience an impact on their quality of life both in the pre- and postoperative period, whether they undergo partial or total thyroidectomy^(^2,3^)^.

The patient’s perception of their health status and the use of scientifically validated tools and instruments are configured as pillars of evidence-based health practice^(^4^)^. In the clinical context presented, data collection takes place through the analysis of subjective information. Thus, when considering the possible changes resulting from thyroidectomy and the processes of planning and therapeutic intervention, the importance of validating the patient’s self-perception regarding vocal parameters stands out, such as the coping strategies adopted for dysphonia and the measurement of vocal handicap.

When facing dysphonia, the individual may adopt strategies to deal with the change and control the resulting stress, which may focus on the problem, aiming to modify the psychological state and the source of stress, or on the emotion, trying to regulate the emotional stress caused by the stressor^(^5^)^. Such strategies minimize physical, emotional, and psychological pressure, resulting in better psychosocial adjustment, quality of life, and mental balance^(^6^)^. However, the literature is not clear about the coping strategies used by patients undergoing thyroidectomy, regardless of the moment. And this understanding, through appropriate instruments, allows the implementation of more assertive and individualized speech therapy care and rehabilitation actions centered on the patient’s needs.

The self-perception of vocal handicap may be greater in patients undergoing partial thyroidectomy^(^7^)^, vary at different times of treatment, and increase by about 20% post-thyroidectomy compared to the preoperative period^(^8-10^)^. Up to two weeks after thyroidectomy, there may be an increase in vocal handicap and a decrease from the second week up to six months, reaching normality thresholds around 1 year after thyroidectomy^(^8^)^. Thus, data from longitudinal studies may help in understanding the temporal patterns and self-perception of vocal handicap in patients undergoing thyroidectomy.

In addition to the aforementioned aspects, the presence of emotional symptoms such as anxiety and depression plays a central role in the context of thyroid diseases, may directly alter the patient’s quality of life, their response and adherence to treatment, as well as lead to the persistence of observed clinical signs^(^7,11-13^)^. Investigating the presence of these symptoms at different times of treatment is essential not only to understand the relationship with vocal aspects but also to identify patients who need targeted and individualized therapeutic support, promoting more effective and assertive interventions.

Given the above, the present study intends to analyze coping strategies in dysphonia, vocal handicap, and symptoms of anxiety/depression in patients undergoing thyroidectomy at three times: preoperative, recent postoperative (maximum 01 week), and 3 months post-thyroidectomy.

## METHOD

A retrospective, quantitative, and descriptive study was carried out with approval from the Ethics and Research Committee of the institution of origin, under numbers 5.804.357.20 and 5.811.939, and the signing of a confidentiality and secrecy agreement. Data were collected from an already existing database originating from the application of self-assessment protocols and demographic clinical data from medical records collected at three times: preoperative, recent postoperative (maximum 01 week), and 3 months post-thyroidectomy, which are part of the routine of the Speech Therapy outpatient clinic of the Head and Neck Surgery Department of a university hospital.

Patient selection was by convenience according to the outpatient schedule, and patients with a diagnosis of thyroid disease, of both sexes, regardless of age, and undergoing partial or total thyroidectomy were included. Patients with associated neurological diseases, laryngeal alterations, and endolaryngeal signs of laryngopharyngeal reflux, visualized by videolaryngoscopy, which is part of the head and neck surgeon’s consultation, such as hyperemia and edema of the posterior third of the glottic and interarytenoid region, were excluded, as well as patients with thyroid hormonal alterations obtained by measuring TSH (thyroid-stimulating hormone) and free T4 in the blood, routinely requested in the outpatient follow-up of patients and collected from medical records at all three times. The exclusion of these patients is justified by the possible interference of these factors in postoperative vocal function, since it could mask the specific expected effects of thyroidectomy.

Data were collected from the Dysphonia Coping Strategy Protocol (PEED-27)^(^5^)^, Vocal Handicap Index (VHI)^(^12^)^, and the Hospital Anxiety and Depression Scale (HADS)^(^13,14^)^. The PEED-27^(^5^)^ protocol is a self-assessment instrument for coping with dysphonia and identifies how people with voice disorders deal with their voice problem and the strategies used. It is composed of 27 items categorized into two types of coping strategies: problem-focused (PF), which correspond to the efforts used to modify the source of stress (items 2, 4, 7, 8, 11, 13, 14, 24, 25, and 26), and emotion-focused (EF), which characterize attempts to regulate the emotional stress caused by the stressor (items 1, 3, 5, 6, 9, 10, 12, 15, 16, 17, 18, 19, 20, 21, 22, 23, and 27). Scoring is according to the frequency of use of coping strategies: 0 (never), 1 point (almost never), 2 points (sometimes), 3 points (frequently), 4 points (almost always), and 5 points (always). The total score corresponds to the simple sum and varies from 0 (zero) to 135 points, in which “0” indicates no use of coping strategies and “135” indicates the use of all strategies in the questionnaire. There is no cutoff point, only averages referring to the dysphonic population (51.8 points) and non-dysphonic population (23.1 points)^(^5^)^.

VHI contains 30 questions covering functional, organic, and emotional aspects related to voice disorders. For each of these domains there are 10 questions, and the score ranges from 0 to 4, with 0 corresponding to “never” and 4 to “always.” The simple sum of the answers determines the value of the domain scores, and the total score is defined by the sum of the three domains, with 120 being the maximum score^(^12^)^. Vocal handicap is considered mild (0 to 30 points), moderate (31 to 60 points), or severe (60 to 120 points)^(^5^)^. The higher the score, the worse the patient’s self-perception of handicap in relation to their voice^(^12^)^, and 50 is considered a high score^(^15^)^.

The HADS self-assesses mood disorders in patients with physical illnesses, contains 14 multiple-choice questions, divided into two subscales, one for anxiety and the other for depression, with seven items each. The global score on each subscale ranges from 0 to 21, while the total score is obtained by summing the two subscales, ranging from 0 to 42. The anxiety and depression scores are categorized as normal (0-7), mild (8–10), moderate (11–14), and severe (15–21). The total score may indicate the general level of emotional disorder, with higher values reflecting greater psychological impairment. The cutoff point of 8 points or more on the total score identifies possible cases of anxiety/depression, and 11 or more points, probable cases of anxiety/depression^(^13,14^)^.

For statistical analysis, descriptive statistics of the studied variables were performed. For comparison of pre- and postoperative moments, the Friedman Test was used. The Conover Post-hoc Test was used to identify at which moments differences occurred. To measure the correlation between the scores of the protocols, the Spearman Correlation Test was used. A significance level of 5% (p-value ≤ 0.05) was adopted. Correlations below 0.50 were considered weak, between 0.50 and 0.7 considered moderate, between 0.70 and 0.90 considered strong, and above 0.90 considered very strong^(^16^)^. All statistical analyses were performed in the R program version 3.6.1.

## RESULTS

Twenty patients with a diagnosis of thyroid disease were included, with a mean age of 54 years (SD±16.9), with a prevalence of female sex (n=17; 85%) and partial thyroidectomy (n=14; 70%).

Regarding PEED-27, a reduction in scores was observed mainly between the preoperative and 3-month postoperative periods, for both EF and PF, with higher scores for EF. However, there was no statistically significant difference between the three moments (Table 1).

**Table 1:** Comparison of the means of PEED-27 and VHI domains at each study time

*PROM*	Domains	Time			p-value
		Pre	Post 1W	Post 3M	
**PEED-27**	EF	17.6	15.6	9.15	0.467
	PF	13.6	11.2	7.5	0.102
	Total	31.3	26.8	16.6	0.351
**VHI**	Emotional	2.15	1.6	2.55	0.272
	Functional	2.85	5.8	5.65	0.703
	Organic	3.75	9.8	7.3	0.102
	Total	8.75	17.2	15.5	0.661

Friedman Test (**p ≤ 0.05)

**Caption:** PROM = Patient-Reported Outcome Measures; PEED-27 = Dysphonia Coping Strategy Protocol; VHI = Vocal Handicap Index; EF = Emotion-Focused; PF = Problem-Focused; Pre = Preoperative; Post 1W = Recent postoperative; Post 3M = 3-month postoperative

Regarding vocal handicap, a mild handicap was observed at all times, with an increase in the recent postoperative period and a reduction after three months, with emphasis on the organic domains and total score. The “organic” domain was the most referenced in the VHI, and regarding the scores of the functional and emotional domains, there was, respectively, an increase followed by stabilization and a decrease followed by an increase in self-reported scores in the preoperative period. However, there was no significant difference (emotional: 0.272; functional: 0.703; organic: 0.102; total: 0.661) between the VHI scores at the three evaluated moments (Table 1).

In HADS, a significant difference (p≤0.05) was observed in the three moments in both subscales, with the greatest difference between preoperative (a) and recent postoperative (b). There was a significant reduction from preoperative to recent postoperative, increasing after three months (c) in all domains. Mild traces of anxiety were observed in the preoperative period, with a reduction in scores in the recent postoperative period and an increase in the three-month postoperative period. In total, a moderate score was observed in the preoperative (13.5) and 3-month postoperative (10.8) periods and mild (8.7) in the recent postoperative period, while the depression subscale remained at levels considered normal. The general emotional state, demonstrated by the total HADS score, was significantly reduced in the recent postoperative period but showed an increase at the three-month follow-up (Table 2).

**Table 2:** Comparison of the means of HADS domains at each study time

*HADS*	Time			p-value
	Pre	Post 1W	Post 3M	
**Anxiety**	8.1ᵇᶜ	5.0ᵃᶜ	6.7ᵃᵇ	0.012**
**Depression**	5.4ᵇᶜ	3.7ᵃ	4.1ᵃ	0.010**
**Total**	13.5ᵇᶜ	8.7ᵃᶜ	10.8ᵃᵇ	0.014**

Friedman Test (**p ≤ 0.05); Conover Post-hoc Test (**p ≤ 0.05)

**Caption:** HADS = Hospital Anxiety and Depression Scale; Preᵃ = Preoperative; Post 1Wᵇ = Recent postoperative; Post 3Mᶜ = 3-month postoperative

A moderate positive correlation was observed, in the recent postoperative period, between all PEED-27 domains and the anxiety subscale. After 3 months, a weak positive correlation was observed between problem-focused coping and anxiety (Table 3).

**Table 3:** Correlations between PEED-27 and VHI domains and HADS subscales at each study time

*PROM*	Domains	*HADS-A*			*HADS-D*			*HADS-T*		
		Pre	Post 1W	Post 3M	Pre	Post 1W	Post 3M	Pre	Post 1W	Post 3M
**PEED-27**	EF	0.014	0.559**	0.401	-0.313	0.399	0.310	-0.194	0.599**	0.382
	PF	-0.060	0.529**	0.462**	-0.372	0.252	0.352	-0.277	0.483**	0.446**
	Total	0.012	0.534**	0.413	-0.340	0.378	0.325	-0.216	0.565**	0.396
**VHI**	Emotional	0.192	0.457**	0.167	0.184	0.302	0.215	0.205	0.491**	0.246
	Functional	0.235	0.426	0.429	0.423	0.319	0.486**	0.398	0.462**	0.487**
	Organic	0.403	0.596**	0.326	0.535**	0.417	0.454**	0.551**	0.623**	0.401
	Total	0.364	0.548**	0.344	0.470**	0.384	0.432	0.489**	0.570**	0.417

Spearman Correlation Test (**p ≤ 0.05)

**Caption:** PROM = Patient-Reported Outcome Measures; PEED-27 = Dysphonia Coping Strategy Protocol; VHI = Vocal Handicap Index; EF = Emotion-Focused; PF = Problem-Focused; HADS = Hospital Anxiety and Depression Scale; HADS-A = anxiety subscale; HADS-D = depression subscale; HADS-T = total subscale

There was no correlation between PEED-27 and the HADS depression subscale. And, between PEED-27 and the total HADS domain, weak/moderate correlations were observed in the recent postoperative period, and a weak positive correlation between problem-focused coping and the total domain in the 3-month postoperative period (Table 3).

In the recent postoperative period, a weak/moderate positive correlation was observed between the emotional, organic domains, and total VHI score and the HADS anxiety subscale. Regarding the HADS depression subscale, there was a moderate positive correlation with the organic domain and a weak correlation with the total VHI score in the recent postoperative period. After 3 months, a weak positive correlation was observed between the “functional” and “organic” domains of the VHI with the HADS depression subscale (Table 3).

Regarding the VHI and the total HADS score, in the preoperative period, moderate and weak positive correlations were observed between the “organic” and “total” domains, respectively. In the recent postoperative period, moderate positive correlations stood out between the “organic” domain of the VHI and the anxiety subscale and the total HADS score, indicating that the perception of handicap is related to levels of anxiety and emotional distress in this period. A weak positive correlation was also identified between the functional domain of the VHI and the total HADS score, both in the recent and 3-month postoperative periods. In addition, in the recent postoperative period, a weak positive correlation was observed between the “emotional” domain of the VHI and the anxiety subscale and the total HADS score, which reinforces the influence of emotional factors on the initial vocal handicap (Table 3).

## DISCUSSION

The vocal self-perception of different vocal parameters before and after thyroidectomy has been reported in the literature^(^8-10,17^)^. Some studies explore the strategies adopted by patients with dysphonia to deal with the vocal disorder, dividing them into problem-focused coping strategies and emotion-focused coping strategies^(^5^)^. It is known that individuals with vocal complaints use up to twice as many coping strategies compared to the general population and that patients undergoing laryngeal surgery use strategies with greater focus on emotion^(^11^)^. However, the scientific literature is scarce regarding the description of coping strategies adopted by patients undergoing thyroidectomy.

In addition, the self-perception of vocal handicap and the psychological state in patients undergoing thyroidectomy is crucial to improving the treatment of thyroid diseases^(^18,19^)^ and, in a certain way, underpins speech-language therapy intervention both pre- and post-thyroidectomy.

The PEED-27 scores showed higher values for the use of EF strategies at all moments of the study. Although the instrument already has more EF items and did not show statistical significance, the highest scores were observed in the preoperative period, corroborating EF coping strategies used in the regulation of stressful emotions^(^20^)^, frequently adopted by patients who present greater psychological stress and low quality of life. On the other hand, the greater the individual’s ability to deal with their vocal problem, the less they will resort to emotion-focused strategies^(^21^)^. Still, psychological distress is a critical determinant of long-term quality of life, and therefore, it is crucial to address emotional aspects in patients with dysphonia after thyroidectomy^(^22^)^.

According to the literature, there is a greater vocal handicap in the postoperative period, with a variation of up to twice the scores between the preoperative and one month postoperative^(^17^)^. In a recent study, 62 patients undergoing thyroidectomy were evaluated between the preoperative, three-week postoperative, and six-month postoperative periods, and self-reported an increase of ten points in the VHI score in 29% of patients and a decrease of ten points in 13% in the first three weeks after the surgical procedure. About six months after surgery, 15% of patients reported an increase of ten points in vocal handicap scores^(^23^)^.

One study^(^9^)^ demonstrated that, after two months of surgery, there is self-reporting of a higher total vocal handicap score with a statistically significant difference compared to the preoperative period. Manipulation of the laryngeal nerve, extent of surgery, post-orotracheal intubation trauma, residual swelling, micro-bleeding, among other factors, may influence the self-reporting of vocal handicap^(^8^)^ and justify the divergence with the literature. However, it is noteworthy that despite preservation of the laryngeal nerve, it is possible that individuals undergoing thyroidectomy still present self-perception of vocal complaints in the long term^(^24^)^.

According to Li et al.^(^24^)^, the VHI scores differ between domains, with 6.2% presenting greater functional impact, 6.8% reporting only organic impact, and 0.3% reporting only emotional distress^(^24^)^. In contrast, in the present study, the organic impact was the most significant, followed by functional, and lastly, emotional. The difference with the study by Li et al.^(^24^)^ may have occurred due to methodological or population differences, which highlights the importance of considering multiple perspectives when assessing the impacts in different domains.

As for the results of the HADS scale, in the anxiety subscale, a significant difference was observed in the preoperative period when compared to the recent postoperative and 3-month postoperative periods, corroborating a previous study that analyzed psychological aspects pre-thyroidectomy^(^25^)^.

Factors such as alertness, anxiety regarding surgery, and lack of information may be relevant for the increase in scores. Regarding the depression subscale, there is no consensus in the literature regarding the self-reporting of depression after surgery. Some authors report a significant reduction in psychiatric symptoms and improvement in mental health shortly after thyroidectomy^(^26^)^. Some studies demonstrate the presence of depressive symptoms immediately after thyroidectomy, persisting up to one-year post-surgery^(^26,27^)^, due to iodine therapy, maintenance of self-medication, and possible short-term metabolic alterations generated by the removal of the thyroid gland^(^27^)^.

There was a significant positive correlation between the domains of PEED-27, VHI, and the HADS scale, highlighting the relevance of the relationship between voice, emotional symptoms, and coping strategies. The relationship between the PEED-27 domains and the HADS anxiety and total subscales in the postoperative periods demonstrates that the greater the self-reporting of anxiety and total, the greater the self-reporting of coping strategies after thyroidectomy. It is important to consider that the total HADS score not only reflects emotional symptoms but may also indicate difficulties in coping with situations that require psychosocial adaptation. Therefore, the need to consider emotional factors in the follow-up of these patients is reinforced^(^28^)^. In addition, the evaluation of coping strategies and individual characteristics, such as personality traits, may be useful in identifying patients who need specific counseling and support, as evidenced in patients with hypothyroidism^(^29,30^)^. It should be emphasized that the role of coping strategies in modulating the perception of vocal handicap, which is relevant since inadequate strategies or those focused exclusively on emotion may amplify the perception of vocal difficulties and worsen emotional symptoms. On the other hand, more adaptive strategies, such as problem-focused coping, may contribute to better psychosocial adaptation over time.

Understanding coping strategies in dysphonia may be useful to identify patients who need specific counseling and support^(^29^)^, since psychological factors seem to play an important role in the development of anxiety symptoms in patients with thyroid diseases^(^30^)^. In addition, emotional aspects such as anxiety and depression may impact the increase of vocal handicap scores^(^21,28^)^ as observed by the significant positive correlations between VHI scores and anxiety and depression symptoms. Thus, it is suggested that the perception of vocal handicap may be more sensitive in patients who present emotional symptoms, pointing to the need for multidisciplinary interventions that combine psychological support and speech therapy rehabilitation. The role of these interventions may be even more relevant in the recent postoperative period, a period of greater emotional and vocal vulnerability, in which adequate coping strategies may mitigate long-term negative impacts.

Therefore, speech-language pathologists must address in their therapeutic planning not only physical issues but also emotional ones when evaluating individuals pre- and post-thyroidectomy^(^28^)^. Strategies such as the use of validated instruments, as used in the present study, to analyze emotional and vocal symptoms, guidance on coping practices, and the promotion of a welcoming space may favor treatment adherence and more rapid and effective rehabilitation. When identifying signs of significant emotional distress, the speech-language pathologist should refer the patient for psychological support, reinforcing the multidisciplinary approach centered on the patient’s needs.

The study was retrospective with analysis of a database from a prospective study. Therefore, we highlight as limitations the small number of participants due to the suspension of the outpatient clinic because of the COVID-19 pandemic at the time of data collection, the difficulty of telephone communication with patients, and the lack of clinical information regarding the indication for thyroidectomy. Despite the limitations, the need for future investigations is pointed out to more comprehensively assess the relationships between coping strategies, vocal handicap, and emotional symptoms, especially in different clinical contexts and with more representative samples. Future studies may also explore integrated therapeutic interventions that combine vocal rehabilitation and emotional support, aiming to improve the quality of life and clinical outcomes of patients undergoing thyroidectomy.

## CONCLUSION

Patients undergoing thyroidectomy showed greater use of emotion-focused coping strategies and self-reported mild vocal handicap, with no difference between the analyzed moments. Mild traces of anxiety were identified in the preoperative period, with a reduction in the recent postoperative period and an increase after three months. Positive correlations from weak to moderate were found between coping strategies and vocal handicap with anxiety symptoms, mainly in the recent postoperative period, during which the most relevant relationships occurred.
