# Supplementary material for: Dysphonia coping strategies, vocal handicap and anxiety/depression symptoms in pre and post-thyroidectomy patients
Source: Codas. 2025 Nov 17;37(6):e20240302. doi: 10.1590/2317-1782/e20240302en (PMC12642777; doi:10.1590/2317-1782/e20240302en)
Supplement: Translation

# Translation: Estratégias de enfrentamento na disfonia, desvantagem vocal e sintomas de ansiedade/depressão em pacientes pré e pós-tireoidectomia

**DOI:** 10.1590/2317-1782/e20240302pt

**Authors:** Marcus Vinicius Conceição Gama, Gabriel Trevizani, Michelle Guimarães, Elma Heitmann

**Affiliations:** 1 Departamento de Fonoaudiologia, Universidade Federal do Espírito Santo – UFES - Vitória (ES), Brasil.; 2 Programa de Pós-graduação em Ciências da Saúde e Comunicação Humana, Universidade Estadual Paulista “Júlio de Mesquita Filho”- Unesp -Marília (SP), Brasil.

**Keywords:** Ansiedade, Depressão, Disfonia, Tireoidectomia, Voz

## Abstract

**Objetivo:**

Analisar estratégias de enfrentamento na disfonia, desvantagem vocal e sintomas de ansiedade/depressão em pacientes submetidos à tireoidectomia.

**Método:**

Estudo retrospectivo com pacientes avaliados no pré-operatório, pós-recente e 3 meses pós-tireoidectomia. Analisou-se os protocolos Estratégia de Enfrentamento na Disfonia (PEED-27), Índice de Desvantagem Vocal (IDV) e Hospital Anxiety and Depression Scale (HADS). Foram excluídos pacientes com alterações de linguagem, neurológicas, laríngeas, hormonais tireoidianas e refluxo.

**Resultados:**

Participaram 20 pacientes, média de idade de 54 anos (DP±16,9), prevalência de sexo feminino (n=17;85%), tireoidectomia parcial (n=14;70%). No PEED-27, houve redução dos escores entre o pré e o pós-recente e o pós 3 meses, com destaque no foco emocional, sem diferença nos momentos. No IDV, o domínio orgânico foi o mais pontuado, com desvantagem leve sem diferença nos momentos. No HADS, houve traços leves de ansiedade no pré-operatório, redução no pós-recente, aumento após três meses e traços normais para depressão. No pré-operatório, houve correlação positiva moderada entre depressão e domínios orgânico e total do IDV. No pós-recente, ansiedade apresentou correlação positiva fraca/moderada com PEED-27 e IDV. Após 3 meses, PEED-27 apresentou correlação positiva moderada com ansiedade e domínio total da HADS e correlação positiva fraca entre os domínios “funcional” e “orgânico” do IDV com depressão.

**Conclusão:**

Os pacientes utilizam estratégias de enfrentamento com foco na emoção, autorreferem desvantagem vocal leve e traços leves de ansiedade no pré-operatório, redução no pós-recente e aumento pós três meses. Houve correlação positiva entre enfrentamento e desvantagem vocal com ansiedade, principalmente no pós-recente, período com relações mais relevantes.

## INTRODUÇÃO

A glândula tireoide é um dos maiores órgãos endócrinos e tem proximidade anatômica com o nervo laríngeo recorrente, um ramo do nervo vago, aumentando o risco de lesão durante o acesso cirúrgico. É responsável pela produção de dois hormônios, a tiroxina (T4) e a tri-iodotironina (T3), os quais agem em praticamente todas as células e são responsáveis pelo controle de diversas partes do metabolismo dos órgãos do corpo humano^(1)^ como no controle dos batimentos cardíacos, peristaltismo intestinal, regulação do ciclo menstrual, fertilidade, temperatura corporal, controle de peso, humor, memória, função cognitiva e controle emocional.

Na presença de nódulos benignos/malignos na glândula tireoide, existem diferentes tratamentos como monitorização, cirurgia, terapia com iodo radioativo, reposição hormonal, radioterapia e quimioterapia. Além da presença dos nódulos, aspectos estruturais, tamanho, localização e outros são levados em consideração quando a indicação é cirúrgica. Os pacientes podem evoluir com alterações de voz/deglutição e apresentar impacto na sua qualidade de vida tanto no pré quanto no pós-operatório, seja ele submetido à tireoidectomia parcial ou total^(2,3)^.

A percepção do paciente frente ao seu estado de saúde e a utilização de ferramentas e instrumentos cientificamente validados configuram-se como pilares da prática em saúde baseada em evidências^(4)^. No contexto clínico apresentado, a obtenção de dados se dá a partir da análise de informações subjetivas. Assim, ao considerar as possíveis alterações consequentes da tireoidectomia e os processos de planejamento e intervenção terapêutica, destaca-se a importância da validação da autopercepção do paciente frente a parâmetros vocais como as estratégias de enfrentamento à disfonia adotadas e a mensuração da desvantagem vocal.

Ao enfrentar a disfonia, o indivíduo pode adotar estratégias para lidar com a mudança e controlar o estresse resultante, as quais podem focar no problema, visando modificar o estado psicológico e a fonte de estresse, ou na emoção ao tentar regular o estresse emocional causado pelo agente estressor^(5)^. Tais estratégias minimizam a pressão física, emocional e psicológica, resultando em melhor ajuste psicossocial, qualidade de vida e equilíbrio mental^(6)^. Contudo, a literatura não é clara sobre as estratégias de enfrentamento utilizadas por pacientes submetidos à tireoidectomia, independente do momento. E esta compreensão, por meio de instrumentos adequados, permite a implementação de ações de cuidado e reabilitação fonoaudiológica mais assertiva e individualizada centrada na necessidade do paciente.

A autopercepção da desvantagem vocal pode ser maior em pacientes submetidos à tireoidectomia parcial^(7)^, variar em diferentes momentos do tratamento e aumentar cerca de 20% pós-tireoidectomia em comparação com o pré-operatório^(8-10)^. Até duas semanas após a tireoidectomia, pode haver aumento da desvantagem vocal e queda a partir da segunda semana até seis meses, com alcance dos limiares de normalidade próximo de 1 ano após a tireoidectomia^(8)^. Assim, dados de estudos longitudinais podem auxiliar na compreensão dos padrões temporais e da autopercepção da desvantagem vocal em pacientes submetidos à tireoidectomia.

Além dos aspectos supracitados, a presença de sintomas emocionais, como ansiedade e depressão exercem um papel central no contexto de doenças tireoidianas, podem alterar diretamente a qualidade de vida do paciente, sua resposta e adesão ao tratamento além de levar à permanência dos sinais clínicos observados^(7,11-13)^. Investigar a presença desses sintomas em diferentes momentos do tratamento é fundamental não apenas para compreender a relação com os aspectos vocais, mas também para identificar pacientes que necessitam de suporte terapêutico direcionado e individualizado, promovendo intervenções mais eficazes e assertivas.

Diante do exposto, o presente estudo pretende analisar as estratégias de enfrentamento na disfonia, a desvantagem vocal e os sintomas de ansiedade/depressão em pacientes submetidos à tireoidectomia em três momentos: pré-operatório, pós-operatório recente (máximo 01 semana) e 3 meses pós-tireoidectomia.

## MÉTODO

Foi realizado um estudo retrospectivo, quantitativo e descritivo com aprovação do Comitê de Ética e Pesquisa da instituição de origem, sob os números 5.804.357.20 e 5.811.939, e assinatura de termo de sigilo e confidencialidade. Os dados foram coletados de um banco de dados já existente oriundos da aplicação de protocolos de autoavaliação e de dados clínicos demográficos dos prontuários coletados em três momentos: pré-operatório, pós-operatório recente (máximo 01 semana) e 3 meses pós-tireoidectomia e que fazem parte da rotina do ambulatório de Fonoaudiologia, do Setor de Cirurgia de Cabeça e Pescoço de um hospital universitário.

A seleção dos pacientes foi por conveniência de acordo com a agenda ambulatorial e foram incluídos pacientes com diagnóstico de doença tireoidiana, de ambos sexos, independentemente da idade e submetidos à tireoidectomia parcial ou total. Foram excluídos pacientes com doenças neurológicas associadas, alterações laríngeas e sinais endo laríngeos de refluxo laringofaríngeo, visualizados por meio de videolaringoscopia, a qual faz parte da consulta do cirurgião de cabeça e pescoço, como presença de hiperemia e edema de terço posterior da região glótica e interaritenóidea, pacientes com alterações hormonais tireoidianas obtidas pela dosagem de TSH (hormônio tireoestimulante) e T4 livre no sangue, solicitadas rotineiramente no seguimento ambulatorial dos pacientes e colhidas dos prontuários em todos os três momentos. A exclusão desses pacientes justifica-se pela possível interferência desses fatores na função vocal pós-operatória, uma vez que poderia mascarar os efeitos específicos esperados da tireoidectomia.

Foram coletados dados do Protocolo de Estratégia de Enfrentamento na Disfonia (PEED-27)^(5)^, Índice de Desvantagem Vocal (IDV)^(12)^ e da Escala Hospitalar de Ansiedade e Depressão (HADS)^(13,14)^. O protocolo PEED-27^(5)^ é um instrumento de autoavaliação do enfrentamento nas disfonias e identifica como as pessoas com distúrbios vocais enfrentam o seu problema de voz e suas estratégias utilizadas. É composto por 27 itens categorizados em dois tipos de estratégias de enfrentamento: com foco no problema (FP), que correspondem aos esforços utilizados para a modificação da fonte de estresse (itens 2, 4, 7, 8, 11, 13, 14, 24, 25 e 26) e com foco na emoção (FE), que caracterizam as tentativas de regulação do estresse emocional causado pelo agente estressor (itens 1, 3, 5, 6, 9, 10, 12, 15, 16, 17, 18, 19, 20, 21, 22, 23 e 27). A pontuação é de acordo com a frequência de utilização das estratégias de enfrentamento: 0 (nunca), 1 ponto (quase nunca), 2 pontos (às vezes), 3 pontos (frequentemente), 4 pontos (quase sempre) e 5 pontos (sempre). O escore total corresponde à somatória simples e varia de 0 (zero) a 135 pontos, no qual “0” indica nenhum uso de estratégias de enfrentamento e “135” indica o uso de todas as estratégias do questionário. Não há um ponto de corte, apenas médias referentes à população disfônica (51,8 pontos) e não disfônica (23,1 pontos)^(5)^.

O IDV contém 30 questões que abrangem aspectos funcionais, orgânicos, e emocionais relacionados aos distúrbios da voz. Para cada um destes domínios há 10 questões e a pontuação varia de 0 a 4, sendo 0 correspondente a “nunca” e 4 a “sempre”. O valor dos escores dos domínios é determinado pela somatória simples das respostas, e o escore total é definido pela somatória dos três domínios, sendo 120 a pontuação máxima^(12)^. A desvantagem vocal é considerada leve (0 a 30 pontos), moderada (31 a 60 pontos) ou intensa (60 a 120 pontos)^(5)^. Quanto maior a pontuação, pior a autopercepção de desvantagem do paciente em relação à sua voz^(12)^ e 50 é considerado um escore alto^(15)^.

A *HADS* autoavalia transtornos do humor em pacientes com doenças físicas, contém 14 questões do tipo múltipla escolha, divididas em duas subescalas, uma para ansiedade e outra para depressão, com sete itens em cada. A pontuação global em cada subescala varia entre zero e 21, enquanto o escore total é obtido pela soma das duas subescalas, variando entre zero e 42. Os escores de ansiedade e depressão são categorizados em normal (0-7), leve (8–10), moderado (11–14) e grave (15–21). O escore total pode indicar o nível geral de transtorno emocional, com valores mais altos refletindo maior comprometimento psicológico. O ponto de corte de 8 pontos ou mais no escore total identifica casos possíveis de ansiedade/depressão e 11 ou mais pontos, casos prováveis de ansiedade/depressão^(13,14)^.

Para análise estatística, foi realizada estatística descritiva das variáveis estudadas. Para comparação dos momentos pré e pós-cirurgia, foi utilizado o Teste de Friedman. O Teste Post-hoc de Conover foi utilizado para identificar em quais momentos ocorreram diferenças. Para medir a correlação entre as pontuações dos protocolos foi utilizado o Teste de Correlação de Spearman. Adotou-se um nível de significância de 5% (p-valor ≤ 0,05). Correlações abaixo de 0,50 foram consideradas fracas, entre 0,50 e 0,7 consideradas moderadas, entre 0,70 e 0,90 consideradas fortes e acima de 0,90 consideradas muito fortes^(16)^. Todas as análises estatísticas foram realizadas no programa R versão 3.6.1.

## RESULTADOS

Foram incluídos 20 pacientes com diagnóstico de doença tireoidiana, com média de idade de 54 anos (DP±16,9), com prevalência de sexo feminino (n=17; 85%) e tireoidectomia parcial (n=14; 70%).

Quanto ao PEED-27, foi observado redução dos escores principalmente entre o pré-operatório e o pós 3 meses, tanto para FE quanto para o FP com escores mais altos para o FE. Porém, não houve diferença estatisticamente significativa entre os três momentos (Tabela 1).

**Tabela 1:** Comparação das médias dos domínios do PEED-27 e IDV em cada momento do estudo

*PROM*	Domínios	Momento			p-valor
		Pré	Pós 1S	Pós 3M	
**PEED-27**	FE	17,6	15,6	9,15	0,467
	FP	13,6	11,2	7,5	0,102
	Total	31,3	26,8	16,6	0,351
**IDV**	Emocional	2,15	1,6	2,55	0,272
	Funcional	2,85	5,8	5,65	0,703
	Orgânico	3,75	9,8	7,3	0,102
	Total	8,75	17,2	15,5	0,661

Teste de Friedman (**p ≤ 0,05)

**Legenda:***PROM* = *Patient-Reported Outcome Measures*; PEED-27 = Protocolo de Estratégias de Enfrentamento na Disfonia; IDV = Índice de Desvantagem Vocal; FE = Foco na Emoção; FP = Foco no Problema; Pré = Pré-Operatório; Pós 1S = Pós-operatório recente; Pós 3M = Pós-operatório 3 meses

Em relação à desvantagem vocal, foi observada desvantagem leve em todos os momentos, com aumento no pós-operatório recente e redução após três meses com destaque para os domínios orgânico e escore total. O domínio “orgânico” foi o mais referenciado do IDV, e em relação aos escores dos domínios funcional e emocional, observa-se, respectivamente, aumento seguido de estabilização e uma diminuição seguida de aumento dos escores autorreferidos no momento pré-operatório. Porém, não houve diferença significativa (emocional: 0,272; funcional: 0,703; orgânico: 0,102; total: 0,661) entre os escores do IDV nos três momentos avaliados (Tabela 1).

Na *HADS* foi observada diferença significativa (p≤0,05), nos três momentos, nas duas subescalas, com maior diferença entre o pré (a) e pós-operatório recente (b). Houve redução significativa do pré-operatório para o pós-operatório recente, aumentando após três meses (c) em todos os domínios. Foi observado traços leves de ansiedade no pré-operatório, com redução de escores no pós-operatório recente e aumento no pós três meses. No total, foi observado escore moderado no pré (13,5) e pós 3 meses (10,8) e leve (8,7) no pós-operatório recente, enquanto a subescala de depressão permaneceu em níveis considerados normais. O estado emocional geral, demonstrado pelo escore total da *HADS*, reduziu significativamente no pós-operatório recente, mas apresentou um aumento no seguimento de três meses (Tabela 2).

**Tabela 2:** Comparação das médias dos domínios do *HADS* em cada momento do estudo

*HADS*	Momento			p-valor
	Pré	Pós 1 S	Pós 3M	
**Ansiedade**	8,1ᵇᶜ	5,0ᵃᶜ	6,7ᵃᵇ	0,012**
**Depressão**	5,4ᵇᶜ	3,7ᵃ	4,1ᵃ	0,010**
**Total**	13,5ᵇᶜ	8,7ᵃᶜ	10,8ᵃᵇ	0,014**

Teste de Friedman (**p ≤ 0,05); Teste Post-hoc de Conover (**p ≤ 0,05)

**Legenda:***HADS* = Escala Hospitalar de Ansiedade e Depressão; Préᵃ = Pré operatório; Pós 1Sᵇ = Pós operatório recente; Pós 3Mᶜ = Pós três meses

Foi observada correlação positiva moderada, no pós-operatório recente, entre todos os domínios do PEED-27 e a subescala ansiedade. Após 3 meses, observou-se correlação positiva fraca entre foco no problema e a ansiedade (Tabela 3).

**Tabela 3:** Correlações entre os domínios do PEED-27 e IDV e as subescalas da *HADS* em cada momento do estudo

*PROM*	Domínios	*HADS-A*			*HADS-D*			*HADS-T*		
		Pré	Pós 1S	Pós 3M	Pré	Pós 1S	Pós 3M	Pré	Pós 1S	Pós 3M
**PEED-27**	FE	0,014	0,559**	0,401	-0,313	0,399	0,310	-0,194	0,599**	0,382
	FP	-0,060	0,529**	0,462**	-0,372	0,252	0,352	-0,277	0,483**	0,446**
	Total	0,012	0,534**	0,413	-0,340	0,378	0,325	-0,216	0,565**	0,396
**IDV**	Emocional	0,192	0,457**	0,167	0,184	0,302	0,215	0,205	0,491**	0,246
	Funcional	0,235	0,426	0,429	0,423	0,319	0,486**	0,398	0,462**	0,487**
	Orgânico	0,403	0,596**	0,326	0,535**	0,417	0,454**	0,551**	0,623**	0,401
	Total	0,364	0,548**	0,344	0,470**	0,384	0,432	0,489**	0,570**	0,417

Teste de Correlação de Spearman (**p ≤ 0,05)

**Legenda:***PROM* = *Patient-Reported Outcome Measures*; PEED-27 = Protocolo de Estratégias de Enfrentamento na Disfonia; IDV = Índice de Desvantagem Vocal; FE = Foco na emoção; FP = Foco no problema; HADS = Escala Hospitalar de Ansiedade e Depressão; *HADS-A* = subescala ansiedade; *HADS-D* = subescala de depressão; *HADS-T* = subescala total

Não houve correlação entre o PEED-27 e a subescala de depressão da *HADS*. E, entre o PEED-27 e o domínio total da *HADS*, houve correlações fracas/moderadas no pós-operatório recente, e correlação positiva fraca entre foco no problema e o domínio total no período de 3 meses após a tireoidectomia (Tabela 3).

No pós-operatório recente, foi observada correlação positiva fraca/moderada entre os domínios emocional, orgânico e escore total do IDV e a subescala ansiedade. Em relação à subescala de depressão da *HADS*, houve correlação positiva moderada com o domínio orgânico e correlação fraca com o escore total do IDV no pós-operatório recente. Após 3 meses foi observada correlação positiva fraca entre os domínios “funcional” e “orgânico ” do IDV com a subescala de depressão da *HADS* (Tabela 3).

Em relação ao IDV e o escore total da *HADS*, no pré-operatório foram observadas correlações positivas moderada e fraca entre os domínios “orgânico” e “total”, respectivamente. No pós-operatório recente, destacaram-se correlações positivas moderadas entre o domínio “orgânico” do IDV e as subescalas de ansiedade e o escore total da *HADS*, indicando que a percepção de desvantagem se relaciona com níveis de ansiedade e sofrimento emocional nesse período. Também foi identificada correlação positiva fraca entre o domínio funcional do IDV e o escore total da *HADS* tanto no pós-recente quanto pós 3 meses. Além disso, no pós-recente, foi observada correlação positiva fraca entre o domínio “emocional” do IDV e as subescalas de ansiedade e o total da *HADS*, o que reforça a influência de fatores emocionais na desvantagem vocal inicial (Tabela 3).

## DISCUSSÃO

A autopercepção vocal de diferentes parâmetros vocais antes e depois da tireoidectomia vem sendo relatada na literatura^(8-10,17)^. Há pesquisas que exploram as estratégias adotadas por pacientes com disfonia para lidar com o distúrbio vocal, dividindo-as em estratégias de enfrentamento centradas no problema e estratégias de enfrentamento centradas na emoção^(5)^. Sabe-se que sujeitos com queixas vocais utilizam até o dobro de estratégias de enfrentamento comparados à população em geral e que pacientes submetidos à cirurgia laríngea utilizam estratégias com maior foco na emoção^(11)^. Contudo, a literatura científica é escassa quanto à descrição de estratégias de enfrentamento adotados por pacientes submetidos à tireoidectomia.

Além disso, a autopercepção da desvantagem vocal e o estado psicológico em pacientes submetidos à tireoidectomia é crucial para aprimorar o tratamento de doenças tireoidianas^(18,19)^ e, de certa forma, embasa a intervenção fonoaudiológica tanto pré quanto pós-tireoidectomia.

Os escores do PEED-27 mostraram valores mais altos para o uso de estratégias de FE em todos os momentos do estudo. Embora o instrumento já possua mais itens com FE e não tenha apresentado significância estatística, os maiores escores foram observados no pré-operatório, corroborando as estratégias de enfrentamento com FE utilizadas na regulação de emoções estressantes^(20)^, frequentemente adotadas por pacientes que apresentam maior estresse psicológico e baixa qualidade de vida. Por outro lado, quanto maior for a habilidade do indivíduo em lidar com seu problema vocal, menos ele recorrerá a estratégias centradas na emoção^(21)^. Ainda assim, o sofrimento psicológico é um determinante crítico da qualidade de vida a longo prazo, e por isso é crucial abordar os aspectos emocionais nos pacientes com disfonia pós-tireoidectomia^(22)^.

Segundo a literatura, há maior desvantagem vocal no pós-operatório, com variação de até o dobro dos escores entre o pré e um mês de pós-operatório^(17)^. Em um estudo recente, 62 pacientes submetidos à tireoidectomia foram avaliados entre o pré, pós-três semanas e pós seis meses e autorreferiram acréscimo de dez pontos no escore do IDV em 29% dos pacientes e decréscimo de dez pontos em 13% nas três primeiras semanas após o procedimento cirúrgico. Por volta de seis meses após a cirurgia, 15% dos pacientes relataram acréscimo de dez pontos nos escores de desvantagem vocal^(23)^.

Um estudo^(9)^ demonstrou que, após dois meses de cirurgia, há autorreferência de maior escore total de desvantagem vocal com diferença estatisticamente significativa para o pré-operatório. A manipulação do nervo laríngeo, extensão da cirurgia, trauma pós-intubação orotraqueal, inchaço residual, micro sangramentos, dentre outros fatores, podem influenciar a autorreferência de desvantagem vocal^(8)^ e justificar a divergência com a literatura. No entanto, salienta-se que apesar da preservação do nervo laríngeo, é possível que os indivíduos submetidos à tireoidectomia ainda apresentem autopercepção de queixas vocais a longo prazo^(24)^.

De acordo com Li et al.^(24)^, os escores do IDV diferem entre os domínios, em que 6,2% apresentaram maior impacto funcional, 6,8% relataram apenas impacto orgânico e 0,3% relataram apenas sofrimento emocional^(24)^. Em contraste, no presente estudo o impacto orgânico foi o mais significativo, seguido pelo funcional e, por último, o emocional. A diferença com o estudo de Li et al.^(24)^, pode ter ocorrido por diferenças metodológicas ou populacionais, o que ressalta a importância de considerar múltiplas perspectivas ao avaliar os impactos em diferentes domínios.

Quanto aos resultados da escala *HADS*, na subescala ansiedade, observou-se diferença significativa no pré, quando comparado ao pós-operatório recente e pós 3 meses, corroborando um estudo prévio que analisou aspectos psicológicos pré-tireoidectomia^(25)^.

Fatores como o estado de alerta, ansiedade em relação à cirurgia e falta de informação podem ser relevantes para o aumento dos escores. Quanto à subescala depressão, não há consenso na literatura em relação à autorreferência de depressão após a cirurgia. Há autores que relatam redução significativa de sintomas psiquiátricos e melhora da saúde mental logo após a tireoidectomia^(26)^. Alguns estudos demonstram a presença de sintomas depressivos imediatamente após a tireoidectomia, com permanência até um ano pós-cirurgia^(26,27)^, devido iodoterapia, manutenção de automedicação e eventuais alterações metabólicas de curto prazo geradas pela retirada da glândula tireoide^(27)^.

Houve correlação significativa positiva entre os domínios do PEED-27, do IDV e da escala *HADS* destacando a relevância da relação entre voz, sintomas emocionais e estratégias de enfrentamento. A relação entre os domínios do PEED-27 e as subescalas ansiedade e total da *HADS* nos períodos pós-operatório demonstra que quanto maior a autorreferência de ansiedade e total, maior a autorreferência de estratégias de enfrentamento após a tireoidectomia. É importante considerar que o escore total da *HADS* não apenas reflete sintomas emocionais, mas também pode indicar dificuldades no enfrentamento de situações que exigem adaptação psicossocial. Por isso, reforça-se a necessidade de considerar fatores emocionais no acompanhamento desses pacientes^(28)^. Além disso, a avaliação das estratégias de enfrentamento e de características individuais, como traços de personalidade, pode ser útil na identificação de pacientes que necessitam de aconselhamento e suporte específicos, como evidenciado em pacientes com hipotireoidismo^(29,30)^. Salienta-se sobre o papel das estratégias de enfrentamento na modulação da percepção de desvantagem vocal, o que é relevante pelo fato das estratégias inadequadas ou focadas exclusivamente na emoção poderem amplificar a percepção de dificuldades vocais e piorar os sintomas emocionais. Por outro lado, estratégias mais adaptativas, como foco no problema, podem contribuir para uma melhor adaptação psicossocial ao longo do tempo.

A compreensão das estratégias de enfrentamento nas disfonias pode ser útil para identificar pacientes que necessitam de aconselhamento e apoio específicos^(29)^, uma vez que fatores psicológicos parecem desempenhar um papel importante no desenvolvimento de sintomas de ansiedade em pacientes com doenças de tireoide^(30)^. Além disso, aspectos emocionais como a ansiedade e a depressão podem impactar no aumento dos escores de desvantagem vocal^(21,28)^ como observada pelas correlações positivas significativas entre os escores do IDV e sintomas de ansiedade e depressão. Assim, sugere-se que a percepção de desvantagem vocal pode ser mais sensível em pacientes que apresentam sintomas emocionais, apontando para a necessidade de intervenções multidisciplinares que combinem suporte psicológico e reabilitação fonoaudiológica. O papel dessas intervenções pode ser ainda mais relevante no pós-operatório recente, um período de maior vulnerabilidade emocional e vocal, em que as estratégias adequadas de enfrentamento podem mitigar impactos negativos de longo prazo.

Portanto, é essencial que fonoaudiólogos abordem em seus planejamentos terapêuticos não apenas as questões físicas, mas também as emocionais, ao avaliar indivíduos pré e pós-tireoidectomia^(28)^. Estratégias como o uso de instrumentos validados, como os utilizados no presente estudo, para analisar sintomas emocionais e vocais, a orientação sobre práticas de enfrentamento e a promoção de um espaço de acolhimento podem favorecer a adesão ao tratamento e uma reabilitação mais rápida e eficaz. Ao identificar sinais de sofrimento emocional significativo, o fonoaudiólogo deve realizar o encaminhamento para suporte psicológico, reforçando a abordagem multidisciplinar centrada nas necessidades do paciente.

O estudo foi retrospectivo com análise de um banco de dados de um estudo prospectivo. Diante disso, destacamos como limitações o pequeno número de participantes devido à suspensão do ambulatório pela pandemia por COVID-19 na época da coleta de dados, a dificuldade de comunicação por telefone com os pacientes e a falta de informações clínicas referentes à indicação da tireoidectomia. Apesar das limitações, aponta-se a necessidade de investigações futuras para avaliar de forma mais abrangente as relações entre estratégias de enfrentamento, desvantagem vocal e sintomas emocionais, especialmente em diferentes contextos clínicos e com amostras mais representativas. Estudos futuros podem também explorar intervenções terapêuticas integradas que combinem reabilitação vocal e o suporte emocional, visando melhorar a qualidade de vida e os resultados clínicos de pacientes submetidos à tireoidectomia.

## CONCLUSÃO

Os pacientes submetidos à tireoidectomia apresentaram maior uso de estratégias de enfrentamento com foco na emoção e autorreferem desvantagem vocal leve, sem diferença entre os momentos analisados. Foram identificados traços leves de ansiedade no pré-operatório, com redução no pós-operatório recente e aumento pós três meses. Houve correlações positivas de fraca a moderada entre as estratégias de enfrentamento e a desvantagem vocal com os sintomas de ansiedade, principalmente no pós-operatório recente, período no qual houve as relações mais relevantes.
